# SIV escape mutants in rhesus macaques vaccinated with NEF-derived lipopeptides and challenged with pathogenic SIVmac251

**DOI:** 10.1186/1743-422X-3-65

**Published:** 2006-08-31

**Authors:** Pascale Villefroy, Franck Letourneur, Zoe Coutsinos, Lorenzo Mortara, Christian Beyer, Helene Gras-Masse, Jean-Gerard Guillet, Isabelle Bourgault-Villada

**Affiliations:** 1 Institut Cochin, Département d'Immunologie, Hôpital Cochin, 27, rue du Faubourg Saint-Jacques, Paris, F-75014, France; 2 INSERM U567, Paris, F-75014, France; 3 CNRS UMR 8104, Paris, F-75014, France; 4 Université Paris 5, Faculté de Médecine René Descartes, UM3, F-75014, France; 5 Institut de Virologie de la Faculté de Médecine, 3 rue Koeberlé, Strasbourg, F-67000, France; 6 INSERM U74, Strasbourg, F-67000, France; 7 Université Pasteur de Strasbourg I, Strasbourg, F-67000, France; 8 Institut de Biologie de Lille, Laboratoire Synthèse, Structure et Fonction des Biomolécules, 1 rue du Professeur Calmette, BP 447, F-59021 Lille Cedex, France; 9 URA CNRS 1309, F-59021 Lille Cedex, France; 10 Université de Lille II, F-59021 Lille Cedex, France; 11 Institut Pasteur de Lille, F-59021 Lille Cedex, France; 12 Assistance Publique-Hôpitaux de Paris, Service de Dermatologie, Hôpital Ambroise Paré, 9 avenue Charles de Gaulle, F-92104 Boulogne, France; 13 Université de Versailles Saint Quentin en Yvelines, Versailles Cedex, F-78035, France; 14 Department of Clinical and Biological Sciences, School of Medicine, University of Insubria, Varese, Italy

## Abstract

**Background:**

Emergence of viral variants that escape CTL control is a major hurdle in HIV vaccination unless such variants affect gene regions that are essential for virus replication. Vaccine-induced multispecific CTL could also be able to control viral variants replication. To explore these possibilities, we extensively characterized CTL responses following vaccination with an epitope-based lipopeptide vaccine and challenge with pathogenic SIVmac251. The viral sequences corresponding to the epitopes present in the vaccine as well as the viral loads were then determined in every macaque following SIV inoculation.

**Results:**

In most cases, the emergence of several viral variants or mutants within vaccine CTL epitopes after SIV challenge resulted in increased viral loads except for a single macaque, which showed a single escape viral variant within its 6 vaccine-induced CTL epitopes.

**Conclusion:**

These findings provide a better understanding of the evolution of CD8+ epitope variations after vaccination-induced CTL expansion and might provide new insight for the development of an effective HIV vaccine.

## Background

Several lines of evidence strongly suggest the key role played by human immunodeficiency virus (HIV)- and simian immunodeficiency virus (SIV)-specific cytotoxic T lymphocyte (CTL) responses in the containment of viral replication and of the disease. CTL responses precede antibody production and coincide with clearance of primary viremia [1-3]. Virus plasma levels within the first 3 months of HIV or SIV infection are predictive of clinical evolution and AIDS-free survival [4-6] and in vivo-depletion of CD8+ T cells during primary infection of rhesus macaques increases plasma viral load [7,8]. Recently and for the first time, anti-GAG CTL induced by a vaccine were shown to be capable to control viral load following intravenous pathogenic SIVmac239 challenge [9].

Several reports showed that anti-HIV immunodominant CTL responses select viral variants bearing mutations that diminish MHC class I binding and/or CTL recognition [10-13]. The viral escape hypothesis has been reinforced by a longitudinal study by Evans et al. in a family of MHC-defined monkeys [14]. This study showed that the progressive amino acid changes in T epitopes throughout the course of infection allowed viruses to escape CTL recognition. Nevertheless, a viral mutation in a CTL epitope can alter the fitness of the virus which can partially loose its infectivity and variability [9]. It is then also very important to characterize which viral regions are essential for maintaining good fitness of the virus. Indeed, vaccination inducing CTL directed against the latter regions allows either a viral control by the CTL or the emergence of viral escape mutants with shift of the virus toward a defective virus.

Very few studies addressed the question of SIV escape due to mutations within multiple epitopes recognized by vaccination-induced CTL. Most published reports focused on particular epitopes recognized by vaccine-induced CTL, such as the epitope MamuA1 CM9 in anti-GAG-SIV-immunized macaques [15] or NEF 128–136 [16]. Although a large debate exists on the role of breadth and magnitude of CD8+ CTL responses in the control of viral replication, several groups have demonstrated in HIV-infected humans that broad specific recognition of CD8+ T cell epitopes was associated with favorable outcome [17-19]. In addition, broad CTL responses are frequently observed in long term survivors [20,21].

With the aim to induce multispecific CTL responses, we previously immunized a cohort of 8 macaques with SIV-NEF- and GAG-derived lipopeptides coupled to tetanus toxoid (TT) 830–846 lipopeptide [22]. Seven of these macaques exhibited CD8+ CTL responses. Two of the responding animals had broad multispecific cytotoxic reactivities directed against four and six SIV epitopes, respectively. We now challenged these 8 macaques with pathogenic SIVmac251 and monitored the evolution of viral sequences in epitopic regions recognized by CTL as well as viral load during the first 8 months after SIV inoculation

## Results

### 1- CTL activities after vaccination with lipopeptides

**Figure 1:**
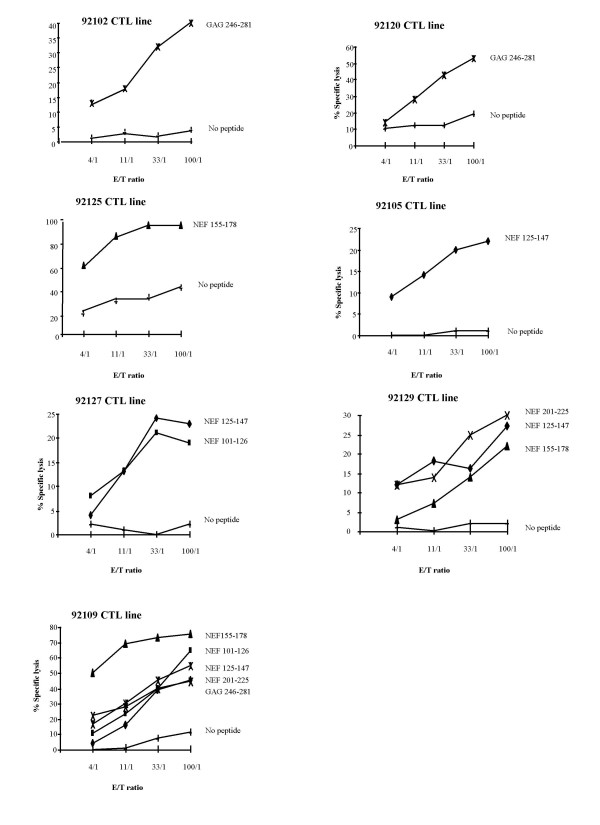
**Cytotoxic activities detected in the 7 responder macaques against long peptides after lipopeptide vaccination**. Only the positive cytotoxic responses against long peptide-sensitized target cells of the responder macaques are shown, all long peptides having been tested in each monkey.

Prior to SIV infection, CTL activities had been induced in seven out of the eight immunized macaques (Figure 1). Two macaques 92109 and 92129 had strong and multi-specific CTL responses that recognized five and three long peptides, respectively. One macaque 92127 had CTL responses against two long peptides with a lower cytotoxic activity. Four other macaques, 92102, 92105, 92120 and 92125, had CTL recognizing a single long peptide and the last macaque, 92117, failed to recognize any peptide.

In order to precisely define the CTL-induced responses, we tested overlapping short peptides spanning the entire sequence of the lipopeptides. Two of the vaccinated macaques, namely 125 and 105, had CTL recognizing a single NEF epitope, NEF 169–178 and NEF 128–136 epitopes, respectively (Table 1). The CTL response of macaque 127 was bi-specific (directed against peptide NEF 116–126 and an unidentified short peptide included in NEF 128–147) and macaque 129 had CTL recognizing 4 epitopes (NEF 128–136, NEF 169–178, NEF 201–211, NEF 211–219). Finally, macaque 109 had CTL that recognized 6 epitopes (NEF 101–110, NEF 116–126, NEF 128–136, NEF 169–178, NEF 215–225, GAG 266–275).

**Table 1:** Epitopic specificities found in 5 immunized macaques

Effector cells^a^		% Specific lysis^c ^at the E/T ratio^d ^of			
Macaque #	Target cells^b^	100:1	33:1	11:1	4:1
92125	None	38	34	25	19
	**NEF 169–178**	**86**^e^	**87**	**70**	**54**

92105	None	22	14	12	2
	**NEF 128–136**	**34**	**24**	**13**	**5**

92127	None		28	21	
	**NEF 116–126**		**43**	**32**	

92129	None	8	3	2	0
	**NEF 128–136**	**46**	**34**	**28**	**9**
	**NEF 201–211**	**22**	**16**	**16**	**5**
	**NEF 211–219**	**19**	**16**	**10**	**4**
	None	52	44	30	
	**NEF 169–178**	**65**	**54**	**33**	

92109	None	14	11	11	5
	**NEF 101–110**		**26**	**22**	**16**
	**NEF 128–136**	**28**	**23**		
	None	41	34	41	27
	**NEF 116–126**	**57**	**45**	**48**	**34**
	None	25	21		
	**NEF 169–178**	**89**	**75**		
	None	14	11		5
	**NEF 215–225**	**41**	**36**		**22**
	None	19	9	7	3
	**GAG -275**	**40**	**24**	**16**	**6**

^a ^CTL cell lines were obtained from PBMCs of the 8 immunized macaques following specific-stimulation with the 7 long peptides *in vitro*.

^b ^Target cells were autologous B-LCLs immortalized by the herpes papio virus and incubated with short peptides (10μM).

^c ^Target cells (5×10^3^) were labeled with 51Cr and incubated for 4 h with various numbers of target cells.

^d ^E/T ratio, effector to target ratio.

^e ^CRT was considered positive if the specific-51Cr release observed against peptide-pulsed target cells exceeded that observed on B-LCLs without peptide by 10% at two E/T ratio.

### 2- Comparison of NEF and GAG CTL epitope sequences included in lipopeptides and in SIVmac251 isolate

Since sequences of the immunizing peptide were derived from the BK-28 SIV clone, epitope variants in the virus inoculum could represent potential viral escape from CTL recognition in lipopeptide-vaccinated macaques [16,23]. We analyzed sequences of viral variants included in the SIVmac251 isolate used for the challenge within the regions present in the lipopeptide vaccine. In the sequenced *gag *gene, we observe no variation within all sequenced SIVmac251 viruses with regards to the peptide sequence GAG 246–281 (data not shown). Similarly, epitopes NEF 116–126 and NEF 169–178 were perfectly conserved (Table 2). In contrast, the other NEF viral CTL epitopic regions varied within the challenge virus. Indeed, within epitopes NEF 211–219 and NEF 215–225, a single amino acid variation was observed in only one of 11 viral sequences (9%) at position 218 (T → A). In epitopes NEF 128–136 and NEF 201–211, two of 11 viral sequences (18%) showed an amino acid change, 136 (A → T) and 202 (K → Q and K → R). Finally, we observed variations of all viral sequences within epitope NEF 101–110, particularly in the first half, with changes at positions 101 (S → P), 102 (V → M), 103 (R → M or R → K), 105 (K → R), and 110 (A → T).

**Table 2:** Comparison between CTL epitope sequences included in lipopeptides and SIV mac251 isolate

	pre-SIV challenge - CTL responses		sequences of SIV mac251 challenge isolate
Macaque #	epitopic peptides	sequences	
125	NEF 169–178	KTFGWLWKLV	KTFGWLWKLV 11/11
105	NEF 128–136	GLEGIYYSA	GLEGIYYS 9/11 GLEGIYY **T **2/11
127	NEF 116–126	AIDMSHFIKEK	AIDMSHFIKEK 11/11
129	NEF 128–136	GLEGIYYSA	GLEGIYYSA 9/11 GLEGIYYS**T **2/11
	NEF 169–178	KTFGWLWKLV	KTFGWLWKLV 11/11
	NEF 201–211	SKWDDPWGEVL	SKWDDPWGEVL 9/11 S**R**WDDPWGEVL 1/11 S**Q**WDDPWGEVL 1/11
	NEF211–219	LAWKFDPTL	LAWKFDPTL 10/11 LAWKFDP**A**L 1/11

109	NEF 101–110	SVRPKVPLRA	**P**V**M**P**R**VPLR**T **8/11 **P**V**M**P**R**VPLRA 1/11 SVRPKVPLR**T **1/11 S**MK**P**R**VPLR**T **1/11
	NEF 116–126	AIDMSHFIKEK	AIDMSHFIKEK 11/11
	NEF 128–136	GLEGIYYSA	GLEGIYYSA 9/11 GLEGIYYS**T **2/11
	NEF 169–178	KTFGWLWKLV	KTFGWLWKLV 11/11
	NEF 215–225	FDPTLAYTYEA	FDPTLAYTYEA 10/11 FDPALAYTYEA 1/11
	GAG -175	WIQLGLQKCV	WIQLGLQKCV 11/11

### 3- Evolution of NEF viral quasispecies within CTL epitopes in macaques following SIV challenge

All vaccinated macaques became infected after SIV challenge. To follow the evolution of NEF epitopic viral sequences, we sequenced the entire *nef *gene in the viruses isolated 35 to 41 weeks after SIV challenge from the vaccinated macaques that had CTL against NEF epitopes (Table 3).

**Table 3:** Evolution of NEF viral quasispecies within CTL epitopes in macaques following SIV challenge

Macaque #	sequences of epitopic peptides		SIV mac251 sequences	post-SIV challenge sequences	weeks
125	NEF 169–178	KTFGWLWKLV	KTFGWLWKLV 11/11	KTFGWLWKLV 10/10	35
105	NEF 128–136	GLEGIYYSA	GLEGIYYSA 9/11 GLEGIYYS**T** 2/11	GLEGIYYSA 5/9 GLEGIYYS**T **4/9	40
127	NEF 116–126	AIDMSHFIKEK	AIDMSHFIKEK 11/11	AIDMSHFIKEK 6/10 AIDMSH**L**IKEK 4/10	40
129	NEF 128–136	GLEGIYYSA	GLEGIYYSA 9/11 GLEGIYYS**T **2/11	GLEGIYYS**T **9/9	35
	NEF 169–178	KTFGWLWKLV	KTFGWLWKLV 11/11	KTFGWLWKLV 9/9	
	NEF 201–211	SKWDDPWGEVL	SKWDDPWGEVL 9/11 S**R**WDDPWGEVL 1/11 S**Q**WDDPWGEVL 1/11	SKWDDPWGEVL 2/9 **AQ**WDDPWGEVL 3/9 **AQ**WDDPWGE**I**L 1/9 **A**KWDDPWGEVL 2/9 S**R**WDDPWGEVL 1/9	
	NEF211–219	LAWKFDPTL	LAWKFDPTL 10/11 LAWKFDP**A**L 1/11	LAWKFDPTL 2/9 LAW**R**FDPTL 3/9 LAWKFD**S**TL 3/9 LAW**R**FD**S**TL 1/9	

109	NEF 101–110	SVRPKVPLRA	**P**V**M**P**R**VPLR**T **8/11 **P**V**MPR**VPLRA 1/11 SVRPKVPLR**T **1/11 S**MK**P**R**VPLR**T **1/11	**P**V**M**P**R**VPLR**T **9/9	41
	NEF 116–126	AIDMSHFIKEK	AIDMSHFIKEK 11/11	AIDMSHFIKEK 9/9	
	NEF 128–136	GLEGIYYSA	GLEGIYYSA 9/11 GLEGIYYS**T **2/11	GLEGIYYSA 9/9	
	NEF 169–178	KTFGWLWKLV	KTFGWLWKLV 11/11	KTFGWLWKLV 9/9	
	NEF 215–225	FDPTLAYTYEA	FDPTLAYTYEA 10/11 FDP**A**LAYTYEA 1/11	FDPTLAYTYEA 9/9	
	GAG -275	WIQLGLQKCV	WIQLGLQKCV 11/11	ND	

Among macaques with anti-NEF induced CTL, the NEF 169–178 sequence was stable (macaque 125). Macaque 105 had a significant increase in 136T viral variants (18%→ 44%). 122L mutants (40%) occurred in macaque 127. In macaque 129, there were many mutations in NEF 201–211 and NEF 211–219 epitopes and emergence of an exclusive 136T variant (100%) in the NEF 128–136 epitope. No variation was evidenced in the NEF 169–178 epitope, as observed in macaque 125 also. As for macaque 109 that lacked detectable cell-associated viremia, viral DNA integrated in PBMC was identified and sequenced. A single viral variant was detected in this latter animal within all the NEF lipopeptide-induced CTL epitopes.

### 4- Monitoring of viral load following SIV challenge

**Figure 2:**
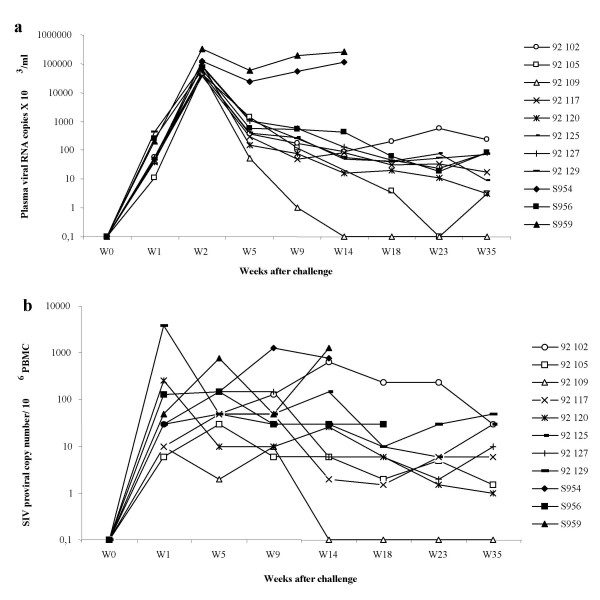
**Evaluation of plasma viral RNA levels and cell-associated viremia in SIV-challenged macaques**. **a- **Plasma viral load in 8 lipopeptide-vaccinated (102, 105, 109, 117, 120, 125, 127, 129) and 3 naive (954, 956, 959) macaques was evaluated up to week 35 post-SIV infection using SIVmac bDNA assay. **b- **Cell-associated viremia was evaluated in 8 lipopeptide-vaccinated (102, 105, 109, 117, 120, 125, 127, 129) and 3 control (954, 956, 959) macaques up to 35 weeks post-SIV inoculation.

High level plasma viral RNA was observed 15 days post-inoculation in all macaques (Figure 2a). The three control animals (954, 956, 959) had a high peak viremia at day 15 post-inoculation. Two of them (954 and 959), with the highest viral load, died at month 4. All but one vaccinated macaque (109), had plasma viral RNA levels that remained high following viremia peak. In contrast, RNA viremia in macaque 109 was consistently undetectable from the third month post-challenge. Macaque 105's plasma viral load was only transiently controlled at week 23.

Cell-associated viremia was measured in all macaques during the same period (Figure 2b). All animals had high cellular viremia except for macaque 109 that had undetectable levels from the third month after SIV-infection. Moreover, median levels of plasma viral RNA and cell associated viremia, evaluated between weeks 9 and 35, were high except for macaque 109 (Table 4).

**Table 4:** Median of plasma viral RNA and cell-associated viremia

	plasma viral RNA	Cell-associated viremia
Macaque #	copies/ml median for weeks 9–35	SIV proviral copy/10^6 ^PBMC median for weeks 9–35

117	33 000	6

102	208 000	230

120	16 000	6

125	48 000	30

105	4 000	5

127	81 000	6

129	57 000	30

109	< 1500	0,1

### 5- Longitudinal follow-up of CTL responses following SIV challenge in macaques 109 and 129

**Figure 3:**
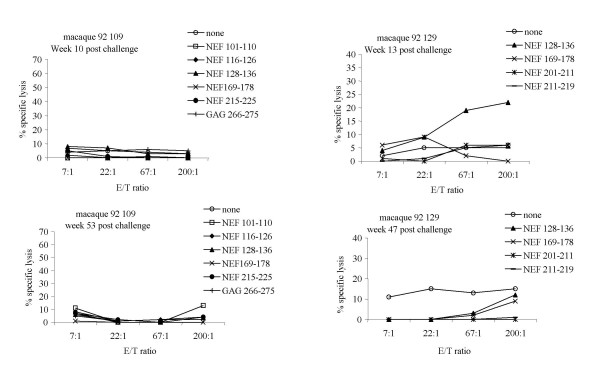
**Post-challenge CTL responses of lipopeptide-vaccinated macaques 109 and 129**, evaluated at weeks 10 and 53 for macaque 109, weeks 13 and 47 for macaque 129. The target cells were autologous B-LCL cells alone (○) or sensitized by short epitopic peptides: NEF 101–110 (□), NEF 116–126 (◆), NEF 128–136 (▲), NEF 169–178 (**X**), NEF 201–211 (*), NEF 211–219 (-), NEF 215–225 (●) and GAG 266–275 (+).

CTL responses were tested both between 10 to 13 weeks and between 47 to 60 weeks after SIV challenge by stimulating PBMC with ConA as described in section methods. CTL responses against the epitopes recognized by lipopeptide-induced CTL (shown in Figure 3a) were no longer detectable in macaque 109 following SIV challenge (Figure 3b). In contrast, macaque 129 had CTL against NEF the 128–136 peptide that were observed at week 13 but became undetectable at week 47 following SIV challenge (Figure 3b).

## Discussion and conclusion

In a previous work, we immunized eight rhesus macaques with SIV-NEF and -GAG lipopeptides combined with a promiscuous TT 830–846 lipopeptide [22]. In the present study, all animals and 3 control macaques were intravenously challenged with pathogenic SIVmac251. This pathogenic viral isolate consisted of a mixture of several viral quasispecies of the *nef *gene that display several differences in particular within the NEF epitopes recognized by lipopeptide-induced CTL.

Five macaques had post-immunization anti-NEF CTL and one of them (macaque 125) had CTL directed only against NEF 169–178, which is perfectly conserved within the challenge SIVmac251 quasispecies. No variation was observed in the sequence of this epitope 35 weeks following SIV challenge. Likewise, this epitope was conserved in both macaques 129 and 109. This result is in accordance with our previous data in another macaque immunized with a similar mixture of NEF- and GAG- lipopeptides [23]. These observations suggest that NEF 169–178 is a stable epitope that is not submitted to the pressure of CTL selection.

Recently, Watkins et al. [24] demonstrated that a high level of CTL against the single GAG 181–189 epitope was not sufficient to control viremia. In rhesus macaques immunized with DNA-*gag-pol*-IL2, emergence of viral mutants occurred in GAG 181–189 after SIV-challenge under the pressure of mono-epitope CTL [25,26]. This viral escape was due to the selection of mutant viral epitopic peptides unable to stably bind to MHC class I molecules, as we have previously shown in lipopeptide-vaccinated macaques within epitope NEF 128–136 [16] and in HIV-infected patients [11]. In addition, the emergence of such viral mutants had no effect on the viral load [16], which suggests no effect on viral fitness.

In the present study, macaque 105 had lipopeptide induced CTL against NEF 128–136, a non-conserved epitope within the pathogenic SIVmac251 isolate, which contains 18% of 136T and 82% of 136A quasispecies. Forty weeks following SIV challenge of this monkey, the percentage of 136T viruses had increased (45%) whereas 136A viruses decreased (55%). The persistence of the two wild type variants within the single vaccine induced CTL epitope did not affect viral replication. The NEF 116–126 epitope recognized by CTL after lipopeptide vaccination in macaque 127 was perfectly conserved in SIV isolate (NEF 116–126) as NEF 169–178 epitope but 122L mutant occurred in 40% of the SIV quasispecies 40 weeks after SIV infection. Nevertheless, the persistence of 60% of wild type viral sequences likely allowed viral replication to remain very high during clinical evolution in this macaque without effect on the high viral fitness.

Three of the 4 epitopes recognized by lipopeptide-induced CTL from macaque 129 were not conserved (NEF 128–136, NEF 201–211 and NEF 211–219) in SIVmac251 isolates. The emergence of the wild type variants 136T (100%) was observed within the CTL epitope NEF 128–136 after SIV challenge. Epitopes NEF 201–211 and 211–219 shifted by acquiring mutations that had no effect on viral load and the persistence of wild type viral sequences (22%) within these epitopes could also have contributed to intense viral replication.

In macaque 109, three of the 6 epitopes recognized by CTL following lipopeptide vaccination, namely epitopes (NEF 116–126, NEF 169–178 and GAG 266–275) were perfectly conserved in SIVmac251 isolates used for the challenge. Interestingly, after challenge, we did not observe any variation within all sequenced NEF epitopes from SIV-infected macaque in particular in epitope NEF 116–126, in contrast to the data in macaque 127. Within epitopes NEF 101–110, NEF 128–136 and NEF 215–225, only one viral variant issued from SIVmac251 was selected and expanded in the absence of emergence of new variations. We hence hypothesize that macaque 109 exerted a selection of few-replicative and non-pathogenic viral variant following SIV challenge. This selection could be the consequence of the vaccine induced CTL. However, we cannot formally exclude the role of an uncontrolled and random process.

CTL responses were evaluated in the two infected macaques 109 and 129, 12 months post-challenge. They were undetectable against all the identified vaccine peptides except in macaque 129. In the latter animal, CTL response against peptide 128–136 disappeared at week 47, following a 100% selection of 136T viral variant as shown in Table 3 and previously observed [16].

CTL obtained following vaccination could play a key role in the control of viremia. Decrease and control of viral load have also been reported in macaques vaccinated with MVA-*gag-pol-env *[27,28], MVA-*nef *[29], MVA-*gag-pol *[30], ALVAC-*gag-pol-env *[31], NYVAC-*gag-pol-env *[32], adeno-*gag *[33], DNA [34,35], a combination of DNA and MVA [36,37] or a prime/boost with DNA/*gag*-Sendai virus [9] and challenged with SIV or SHIV. In these studies, the control of SIV/SHIV replication was clearly related to a high magnitude of CTL recognizing NEF [29] or GAG 181–189 epitope in MamuA1 macaques [30,35], or to the selection of a non pathogenic viral mutant in GAG 206–216 (216S) CTL vaccine epitope [9]. Indeed, viral escape by mutation in an epitope under CTL pressure can also prevent virus replication. Matano et al [9] observed that after vaccination with DNA/gag-Sendai and viral challenge, all macaques that controlled viral replication had a mutation in GAG leading to the substitution of one residue in GAG 206–216 (216S) CTL vaccine epitope by week 5 after challenge. This viral escape variant could have a lower fitness than wild type SIVmac239, indicating that the vaccine-induced CTL could have exerted a strong immune pressure leading to clearance of the wild type pathogenic SIV.

In our study, the emergence of several viral mutants in two macaques (127 and 129) within vaccine CTL epitopes was always associated with the persistence of the wild type virus and therefore was not concomitant with the decrease of viral fitness. The occurrence of an exclusive viral escape variant within several vaccine induced CTL epitopes was observed in only one macaque (109) and could be associated either with a selection of a poor replicative virus or with a control of viral replication by CTL.

These results tentatively bring a clue for a better understanding of SIV control and might provide new insight for the development of an effective HIV vaccine.

## Materials and methods

### Lipopeptides and short peptides

Five peptides from the SIV-NEF protein were synthesized from the sequence of the molecular clone SIV BK-28 (LP1 aa 101–126, LP2 aa 125–147, LP3 aa 155–178, LP4 aa 201–225, and LP5 aa 221–247). Two peptides from the SIV-GAG protein were also produced (LP6 aa 165–195 and LP7 aa 246–281). These selected sequences were identical to those previously reported [38] except for the introduction of a N^ε ^palmitoyl-lysylamide at the C-terminal position. A tetanus toxoid (TT) 830–846 lipopeptide was added to the seven SIV lipopeptides [22]. The lipopeptides were synthesized by solid-phase synthesis as previously described [39]. They were purified to more than 90% homogeneity by reverse-phase HPLC and characterized by amino acid composition and molecular mass determination. In addition, overlapping short peptides spanning the entire sequence of these lipopeptides were synthesized by Neosystem (Strasbourg, France).

### Immunization protocol and virus challenge

Eight rhesus macaques (*Macaca mulatta*) were immunized with SIV-lipopeptides as previously described (three injections at one-month intervals) [22]. They were immunized again 12 and 18 months after the end of the first vaccination cycle. They were challenged intravenously two weeks after the second boost with 10 animal-infectious doses 50 (AID_50_) of the highly pathogenic SIVmac251 isolate, kindly provided by A.M. Aubertin (Strasbourg, France). Three non-vaccinated control macaques received the same challenges. All animal experiments were performed in accordance with European Union guidelines.

### Characterization of CTL responses

The lipopeptide-induced CTL responses were examined after the last mixed-micelle immunization by stimulating macaque PBMCs with a mixture of the seven long free SIV peptides corresponding to the sequences of peptides included in lipopeptides. CTL lines were then tested against autologous B lymphoblastoid cell lines (B-LCL) sensitized by the same long peptides or by short peptides. After SIV challenge, production of SIV antigens by infected CD4+ cells for stimulation of CTL lines, was induced by 14 days stimulation of PBMC with 10 μg/ml concanavalin A (Sigma, St.Louis, Mo.). Interleukine (IL) 2 (10 IU/ml, Roche, Mannheim, Germany) was added on days 3, 7, and 10 and cell concentration was adjusted to 5 × 10^5^/ml twice a week.

### In vitro transformation of B cell lines

B lymphoblastoid cell lines (B-LCL) were generated as previously described [38] and cultured in the same medium as that used for the generation of CTL lines.

### Chromium release test (CRT)

To sensitize target cells by peptides, B-LCL (10^6^) were incubated either overnight or for 1 h with long (10^-5 ^M) or short peptides (10^-6 ^M) at 37°C in a humidified 5% CO2 atmosphere. B-LCL alone served as controls. B-LCL were washed and labeled with 100 μCi Na_2_^51^CrO_4 _(NEN Life Science Products, Courtaboeuf Les Ullis, France) for 1 h, washed twice, and used as target cells. CRT was performed in V-bottomed 96-well microtiter plates. The cytolytic activity of anti-SIV cell lines was measured by mixing 5,000 ^51 ^Cr-labeled target cells with effector cells at various effector cell/target cell (E/T) ratios in a final volume of 200 μl/well. Duplicate wells were seeded for each E/T ratio. Plates were incubated for 4 h at 37°C; 100 μl/well of supernatant was then removed from each well and counted. Spontaneous release was determined by incubating target cells with medium alone; it never exceeded 20% of total ^51^Cr incorporated. Results were expressed as specific Cr release : 100 × experimental counts per minute (cpm)- spontaneous cpm/maximum cpm – spontaneous cpm. The within-sample variation never exceeded 5%. CRT was considered positive when the specific-^51^Cr release observed against peptide-pulsed target cells exceeded that observed with B-LCL alone by 10% at two effector/target (E/T) ratios.

### Measurement of plasma viral RNA levels

SIV-RNA plasma levels were determined by using the SIVmac bDNA assay (Chiron Diagnostics, Emeryville, CA). The detection threshold was 1500 DNA copies per milliliter of plasma.

### Measurement of cell-associated viremia

To quantify cellular viremia, 10^5 ^CEM X 174 cell hybrids (fusion product of human B-cell line 721.174 and human T-cell line) were co-cultured with fivefold serial dilutions of PBMC. Supernatants of 30-day cultures were tested for the presence of RT SIV antigen.

### Sequencing of SIV genes

#### DNA preparation

PBMC were isolated as above and washed in RPMI medium. Aliquots (10^7 ^cells) were incubated overnight at 52°C in 1 ml lysis buffer (10 mM Tris-HCl pH 8.3, 50 mM KCl, 2.5 mM MgCl_2_, 0.45% Tween 20, and 400 μg/ml proteinase K). DNA was extracted with phenol/chloroform and precipitated with ethanol. The pellet was washed with 70% ethanol, dried, resuspended in 10 mM Tris pH 7.5 and quantified by measuring optical densities at 260 nm.

#### Polymerase chain reaction (PCR) amplification

Nested PCR was performed in 100 μl reaction mixtures containing 200 μM of each deoxynucleotide triphosphate (Pharmacia, Uppsala, Sweden), 10 mM Tris-HCl pH 8.3, 50 mM KCl, 1.5 mM MgCl_2_, 2.5 U Taq polymerase (Gibco BRL, Life Technologies, Gaithersburg, MD), and 20 pmol of primer (Genset, Paris, France). The primers used in the first round of PCR were nef1 (5'-AGGCTCTCTGCGACCCTACG-3') and nef2 (5'-AGAACCTCCCAGGGCTCAATCT-3'). VJ11 (5'-ATGGGTGGAGCTATTTCCATG-3') and VJ12 (5'-TTAGCCTTCTTCTAACCTC-3') (encompassing the entire n*ef *gene) were used in the second round. For gag gene, primers used in the first round of PCR were VJ23 (5'-ATGGGCGCGAGAAACTCCGTC-3') and SIVGAGrev (5'- CCCCTGTATCCAATAATACT -3'). 2 nested PCR were used in a second round of PCR with VJ23 and SIVG3 (5' TGTTGTCTGTACATCCACTGGAT 3'), SIVG1 (5' AGCGGCAGAGGAGGAAATTAC 3') and VJ25 (5'-CTACTGGTCTCCTCCAAAG 3') respectively (encompassing the entire gag gene). Each initial reaction contained 1 μg DNA, and 5 μl of the first PCR round were used in the second round. The reactions were carried out in a DNA thermocycler 9600 (Perkin Elmer, Branchburg, NJ) for 40 cycles (1 min at 96°C for the first cycle and 30 sec at 95°C, 30 sec at 55°C and 1 min at 72°C for the subsequent ones) with a final incubation at 72°C for 5 min. Amplified products were visualized on 1.5% agarose gels after staining with ethidium bromide.

#### Reverse transcription-PCR (RT-PCR)

Viral RNA was extracted from 400 μl of the viral stock using 300 μl phenol acid (Appligene Oncor, Illkirch, France) and 300 μl extraction buffer (7 M urea, 0.35 M NaCl, 10 mM Tris-HCl pH 7.5, 10 mM EDTA, 1% SDS). After vortexing and centrifugation, the supernatant was extracted twice with phenol, twice with chloroform, and then ethanol-precipitated with 2 μg of tRNA. Following centrifugation, the RNA pellet was washed with 70% ethanol, dried, and resuspended in 50 μl sterile water. Five μl were reverse-transcribed for one hour at 37°C in 25 μl reaction mixture containing 50 mM Tris-HCl pH 8.3, 75 mM KCl, 3 mM MgCl_2_, 8 mM DTT, 400 μM each dNTP, 50 pmol primers nef2 and nef1, 30 U RNAsin (Promega, Madison, WI), and 200 U Mo-MuLV reverse transcriptase (Gibco BRL). The PCR mix was incubated for 5 min at 90°C, and 5 μl of the cDNA mixture was amplified under the same PCR conditions as above, using VJ11 and VJ12 as primers.

#### Cloning and sequencing

To estimate viral population diversity and eliminate cloning bias, multiple plasmid subclones derived from the same viral template by using endpoint DNA dilution techniques were sequences. The proviral DNA copy number used in each PCR was approximated by duplicate 10-fold serial dilutions of DNA followed by nested PCR capable of amplifying a single provirus (as described above). The highest dilution yielding a positive PCR was used to estimate the proviral copy number. This end-point dilution of all PBMC DNA generated PCR products that were directly sequenced after purification on a Qiaquik column (Qiagen Courtaboeuf, Les Ullis, France). Following purification, 50 ng of the PCR product was ligated overnight at 15°C with 50 ng of pTAG vector (R&D Systems Europe, Abingdon, UK) in 10 μl of buffer containing 50 mM Tris-HCl pH 7.6, 10 mM MgCl_2_, 1 mM ATP, 1 mM DTT, 5% PEG-8000, and 1 U of T4 DNA ligase (Gibco). A volume of 0.1 μl of the ligation product was transferred into *E. coli *TG1, and the few white colonies obtained on Luria Broth (Amersham Pharmacia Biotech, Amersham, UK) plates with ampicillin were selected. DNA was extracted using the Easy Prep Plasmid Prep kit (Pharmacia) and 500 ng were sequenced using the Dye Terminator chemistry on a 373A sequencer (ABI, Perkin Elmer). All sequences were aligned using the SeqEd program.

## Competing interests

The author(s) declare that they have no competing interests.

## Authors' contributions

FL performed all the sequences of SIV *nef *genes. PV and ZC interpreted the results, prepared the tables, figures and efficiently participated to the writing of the manuscript. LM performed the experiments following lipopeptide vaccination. CB measured cell-associated viremia and HGM synthesized the lipopeptides.

IBV designed and coordinated the study and drafted the manuscript. JGG was responsible for the broad design of the study.

All of the authors made meaningful contributions to the process of successive draft versions of the text. All authors read and approved the final manuscript.
